# Genome-Wide Identification and Expression Profiling of DUF221 Gene Family Provides New Insights Into Abiotic Stress Responses in Potato

**DOI:** 10.3389/fpls.2021.804600

**Published:** 2022-01-20

**Authors:** Madiha Zaynab, Jiaofeng Peng, Yasir Sharif, Mohammed Albaqami, Rashid Al-Yahyai, Mahpara Fatima, Muhammad Azhar Nadeem, Khalid Ali Khan, Saqer S. Alotaibi, Ibrahim A. Alaraidh, Hassan O. Shaikhaldein, Shuangfei Li

**Affiliations:** ^1^Shenzhen Key Laboratory of Marine Bioresource and Eco-Environmental Sciences, College of Life Sciences and Oceanography, Shenzhen University, Shenzhen, China; ^2^Instrument Analysis Center, Shenzhen University, Shenzhen, China; ^3^College of Plant Protection, Fujian Agriculture and Forestry University, Fuzhou, China; ^4^Department of Biology, Faculty of Applied Science, Umm Al-Qura University, Makkah, Saudi Arabia; ^5^Department of Plant Sciences, College of Agricultural and Marine Sciences, Sultan Qaboos University, Muscat, Oman; ^6^College of Agriculture, Fujian Agriculture and Forestry University, Fuzhou, China; ^7^Faculty of Agricultural Sciences and Technologies, Sivas University of Science and Technology, Sivas, Turkey; ^8^Research Center for Advanced Materials Science (RCAMS), King Khalid University, Abha, Saudi Arabia; ^9^Unit of Bee Research and Honey Production, Faculty of Science, King Khalid University, Abha, Saudi Arabia; ^10^Faculty of Science, King Khalid University, Abha, Saudi Arabia; ^11^Department of Biotechnology, College of Science, Taif University, Taif, Saudi Arabia; ^12^Botany & Microbiology Department, Science College, King Saud University, Riyadh, Saudi Arabia

**Keywords:** gene family, miRNA, expression, duplication, phylogeny

## Abstract

The domain of the unknown function 221 proteins regulate several processes in plants, including development, growth, hormone transduction mechanism, and abiotic stress response. Therefore, a comprehensive analysis of the potato genome was conducted to identify the deafness-dystonia peptide (DDP) proteins’ role in potatoes. In the present study, we performed a genome-wide analysis of the potato domain of the unknown function 221 (DUF221) genes, including phylogenetic inferences, chromosomal locations, gene duplications, gene structures, and expression analysis. In our results, we identified 10 *DDP* genes in the potato genome. The phylogenetic analysis results indicated that *StDDPs* genes were distributed in all four clades, and clade IV was the largest clade. The gene duplication under selection pressure analysis indicated various positive and purifying selections in *StDDP* genes. The putative stu-miRNAs from different families targeting *StDDPs* were also predicted in the present study. Promoter regions of *StDDP* genes contain different *cis*-acting components involved in multiple stress responses, such as phytohormones and abiotic stress-responsive factors. The analysis of the tissue-specific expression profiling indicated the *StDDPs* gene expression in stem, root, and leaf tissues. We subsequently observed that *StDDP4*, *StDDP5*, *and StDDP8* showed higher expressions in roots, stems, and leaves. *StDDP5* exhibited high expression against heat stress response, and *StDDP7* showed high transcript abundance against salt stress in potatoes. Under abscisic acid (ABA) and indole acetic acid (IAA) treatments, seven *StDDP* genes’ expressions indicated that ABA and IAA performed important roles in immunity response. The expression profiling and real-time qPCR of stems, roots, and leaves revealed *StDDPs’* significant role in growth and development. These expression results of *DDPs* are primary functional analysis and present basic information for other economically important crops.

## Introduction

Almost 70% of annual potential crop yield losses are due to variations in the environment (Leng et al., 2021). Abiotic factors are the major limiting stress factors that affect plants during their vegetative and reproductive growth, resulting in abrupt economic and agricultural losses worldwide (Sade et al., 2018; Hafeez et al., 2021; Li et al., 2021). Salt and drought stress factors are the chief abiotic stress factors affecting geographical plant distribution, threatening food security, and limiting crop production (Muhammad Ahmad et al., 2021). Generally, plants lack a structure that works directly with environmental impacts, but they can respond to climate change (Mahalingam and Fedoroff, 2003; Huang et al., 2021; Raza et al., 2021c). Plants exhibit extensive defense responses at molecular and cellular levels to oppose the cell damage caused by stress factors (Kim et al., 2014; Raza et al., 2021b).

Plant biologists have always been attracted to gene families’ structure, function, and evolutionary model. The interaction and adaptation between the environment and plants are well studied based on the information of these gene families (Zhao et al., 2021). Among them, the domain of unknown function (DUF) proteins are extensively distributed in different plants and restrain at least one extremely conserved domain of DUF (Bateman et al., 2010). Many studies on DUF genes have revealed the importance of DUFs in plants in various functions, one of which is abiotic stress tolerance (Zhang et al., 2021). However, there have been reports of other DUF gene families in many plants. These include the DUF221, DUF810, DUF866, DUF936, and DUF1618 gene families in rice and the DUF581 and DUF724 gene families in Arabidopsis (Zhao et al., 2021). Furthermore, DUF proteins act as integral membrane proteins and are associated with other membrane proteins (Rai et al., 2012). Resistance to DUF-mediated strains has been studied in various plants, while a comprehensive study of the DUF family gene remains to be conducted in other plants. Additionally, in transgenic *Oryza sativa* (rice) and *Arabidopsis thaliana*, *OsSGL* presents significant drought stress tolerance, whereas other diverse stress-responsive genes have been altered significantly in transgenic rice. Xin et al. (2007) reported that in *A. thaliana*, the *AT3g55990* (*ESK1* gene) is associated with the DUF283 family gene, considered a novel negative cold acclimation gene regulator. The *AtCSC1* gene in Arabidopsis (Hou et al., 2014) and its homologous gene in rice are known *OsCA1*, responsible for osmotic regulation (Yuan et al., 2014), *ERD4* is known as a dehydration-responsive gene in *Brassica juncea* (Rai et al., 2012), whereas *DUF27* and *DUF538* have chlorophyll-binding capabilities (Gholizadeh, 2016). Moreover, the superfamily of *DUF283* is important for siRNA processing (in gene silencing) in Arabidopsis (Qin et al., 2010). Another study in *A. thaliana* illustrated the reduced effect of ABA mediation (drought stress gene) after inhibiting the gene expression of both *ATRDUF2* (RING-DUF1117 E3 ubiquitin ligases) (Kim et al., 2012). From rice, two genes (*DUF1644* and *OsSIDP366*) positively regulate the drought and salt stresses by overexpressing *OsSIDP366* in transgenic rice, which enhances tolerance against drought and salinity factors (Guo et al., 2016). In *O. sativa*, other DUF family genes have been related to abiotic stress factors, including *OsDUF810.7* (Li et al., 2018), *DUF1644* (*SIDP361*) (Li et al., 2016), and *DUF966* (*OsDSR2*) (Luo et al., 2014). Three family members of deafness-dystonia peptides (DDPs) in *Medicago truncatula* reported dehydration (*MtST2* and *MtST3*) and modulation through nutritional status (*MtST1*) during development (Albornos et al., 2017).

Potato is an important food source and cash crop worldwide (Zaynab et al., 2021c). In 2018, about 368,168,914 tons of potatoes were harvested globally from 17,578,672 ha of land, and over one billion people consume them. Among the potato-producing countries, China holds the first position, producing about 90,321,442 tons of potatoes from 4,813,542 ha of land in 2018 (Rashid et al., 2021). Though, like other plants, potato yield is also at risk to abiotic and biotic stress factors (Zaynab et al., 2021f). The objective of the present research was to characterize the *DDP* genes family across the potato genomes. Thus, based on the putative role of *DDPs* against abiotic stress, we carried out a comprehensive analysis of *DDP* genes in the potato genome against abiotic stress. To date, *DDP* genes expression analysis against abiotic has not been reported in potatoes. We examine the expression profiling of *StDDPs* under phytohormones, salt, and heat stress. These data may potentially explain the validated functional foundation of potato *DUF221* genes and their functions according to growth and development in potatoes under unfavorable/stressful conditions.

## Materials and Methods

### Identification and Phylogenetic Analysis of the StDDP Genes

The Arabidopsis *DDPs* sequences were attained from the TIAR^1^ and used as the query to search the potato *DDP* genes from the Phytozome site^2^. A local BLASTP similarity search was carried out to find the *DDPs* in the *S. tuberosum* genome. The collected gene sequences were subjected to PfamScan and Batch CDD-NCBI search to validate the presence of the Pfam domain (PF14703). The data redundancy was removed, and the identified genes were analyzed for further study. The protein physiochemical properties, including isoelectric point (PI), and molecular weight (MW), were forecasted by Expasy^3^ server.

A phylogenetic tree was constructed to observe evolutionary relationships among *A. thaliana*, *Solanum tuberosum*, and *Solanum lycoperiscum* by MEGA 7 software. The entire protein sequences were arranged by using MUSCLE through 16 iterations. Then, the protein sequence alignment was used to make the phylogenetic tree through the Neighbor-Joining method through 1,000 bootstrap values.

### Chromosomal Location, Synteny, and Selection Pressure Analysis

The information about *StDDPs* was retrieved from the Potato Genome Sequencing Consortium (PGSC). The TBtools software^4^ was used to map the chromosomal positions of *StDDPs*. The comparative synteny analysis was executed to visualize the genome conservation through the Circoletto Tool (tools.bat.infspire.org/circoletto/). Further, the duplicated genes’ coding sequences were arranged by the Muscle program in MEGA 7. The synonymous and non synonymous substitution rates (Ka = No. of nonsynonymous substitution/nonsynonymous site; Ks = No. of synonymous substitution/synonymous site) were calculated by KaKs_ Calculator 2.0 software tool through the exchange rate (*r* = 2.6 × 10^–9^; Li et al., 2019).

### Gene Structure and Conserved Motif Analysis

For the gene structure analysis, we used Gene Structure Display Server, and DDP proteins’ conserved motifs were determined by MEME tool^5^ through the following parameters: optimum width ranges: 6–200; no. of motifs: 20. TB tools software^4^ was used to figure out the distribution of motifs.

### *Cis*-Elements Analysis miRNAs Prediction and Orthologous Genes Identification

To analyze the *cis-*regulatory elements of potato *DDP* genes, the promoter sequences (2,000 bp upstream of the ATG initiation codon) were taken from the *S. tuberosum* genome database in a generic file format. The promoter sequences were scanned with the PlantCARE database^6^. The coding sequence of *StDDPs* was used to identify potential miRNAs targeting the *StDDPs* using the psRNATarget database^7^ with default parameters. The orthologous DDPs proteins in *A. thaliana*, *S. tuberosum*, and *S. lycoperiscum* were identified using orthovenn2 https://orthovenn2.bioinfotoolkits.net/home. Protein sequences of three species were used for analysis. Each species was individually assessed with each other for the identification of orthologous gene clusters.

### Expression Analysis of StDDP Genes

For the *StDDP* genes expression analysis, fragments per kilobase million (FPKM) values in root, stem, and leaf tissues were used. The data was collected and assembled with enormously expressed tissues, including leaves, stems, and roots. The FPKM values were used to illustrate the heat map by using TBtools. The log_2_ normalized values were used to construct the heat maps (Zaynab et al., 2021a).

### Plant Sampling and Material Collection

Potato tubers were obtained from the NARC (National Agricultural Research Center), Islamabad, Pakistan, and planted in the glasshouse under controlled conditions at National Institute for Genomics and Advanced Biotechnology (NIGAB) NARC, Islamabad, Pakistan. Later, 25 days after germination, the roots, stems, and leaves were collected in replicates and for RNA extraction stored in liquid nitrogen.

### RNA Extraction and RT-qPCR Analysis

The total RNA from roots, stems, and leaves was extracted by a quick isolation Ribonucleic acid (RNA) Kit (Huayueyang, Beijing, China) following the manufacture’s protocol. The quality of RNA was assessed through gel electrophoresis using 1% agarose gel. The first cDNA strand was prepared from 0.5 μg RNA. The qRT-PCR was executed in BioRad CFX96 RT-PCR Detection System instrument (BioRad Laboratories) with a 20 μL reaction mixture through SYBR ^®^ Green RT-PCR Master (TOYOBO QPK-210, Shanghai, China) using gene-specific primers. The thermocycler was set according to the given protocol: denaturation at 95°C for 15 s, annealing occurs at 55°C for 15 s, and extension takes to play at 72°C for 15 s.

Target gene amplification was monitored with SYBR Green fluorescence in each cycle. In addition, the qRT-PCR amplification specificity was routinely checked with the melting curve. The data were observed through the 2^–ΔΔCt^ method (Zaynab et al., 2018), while results were represented through relative gene expression level (Zaynab et al., 2021d). Through this analysis report, elongation factor 1 elongation factor 1 - α was considered housekeeping gene. In the whole experimental observation, four technical replicates were conducted (Zaynab et al., 2021b). The sequences of all primers used are mentioned in Supplementary Table 1.

## Results

### Identification and Phylogenetic Analysis of StDDP Genes

**FIGURE 1:**
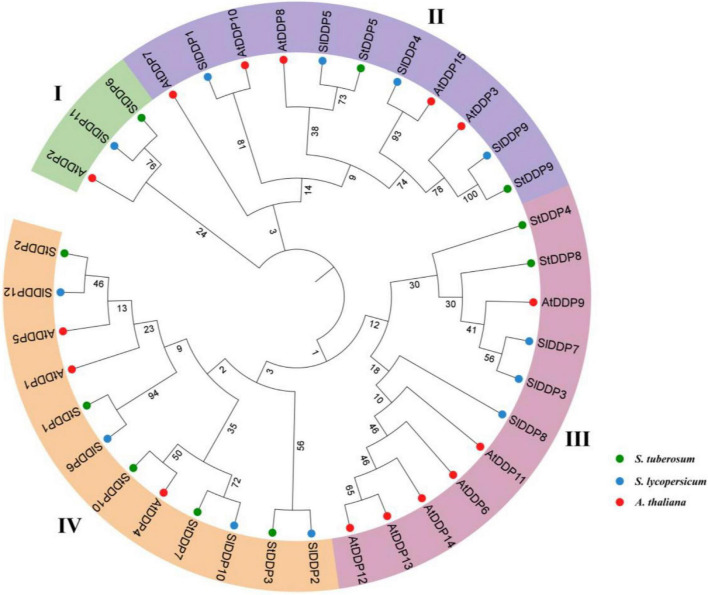
Phylogenetic tree of deafness-dystonia peptide (DDP) proteins from *Arabidopsis, Solanum lycoperiscum*, and *Solanum tuberosum*. The colored arcs indicate different groups. The stars’ colors, represent proteins of *Arabidopsis S. lycoperiscum*, and *S. tuberosum*.

The results showed that 10 *DDP* genes were found after genome-wide identification in the potato genome. Similar genes with diverse transcripts were not considered in this study. Although all the identified genes contain conserved *DDP* domains with Pfam ID (PF14703). The details of 10 *DDPs*, including their molecular weight (MW), chromosome number, isoelectric point (PI), and protein length, are shown in Table 1. Moreover, the protein lengths ranged from 99 to 892 amino acids (aa), the PI was 8.86–9.74, and the MW was from 11.371 to 10.2546 kDa. The evolutionary relationship of *DDPs* was observed through a phylogenetic tree by MEGA7.0 software using the Neighbor-joining method with 1,000 bootstrap values. The phylogenetic tree was classified into four clades. The results of the phylogenetic analysis indicated that *StDDPs* genes were distributed in all four clades and clade IV was the largest clade (Figure 1).

**TABLE 1:** List of identified putative *StDDPs* and their features.

Gene ID	Genomic	CDS	Location	Star-end	No. of amino acids	Molecular weight	PI
StDDP1	7254	2106	ST4.03ch04	66454282..66461536	701	79,307.25	9.5
StDDP2	9609	2655	ST4.03ch12	56091976..56101585	884	99,784.46	9.35
StDDP3	7570	2304	ST4.03ch02	17639083..17646653	767	87,867.64	9.24
StDDP4	6811	1245	ST4.03ch02	36697175..36703986	414	47,703.76	9.4
StDDP5	8295	2151	ST4.03ch02	45947456..45955751	716	81,093.79	8.95
StDDP6	7418	2256	ST4.03ch09	46690336..46697754	751	85,855.55	9.11
StDDP7	8795	2679	ST4.03ch08	48862785..48871580	892	102,546.31	8.86
StDDP8	6811	300	ST4.03ch02	36697175..36703986	99	11,371.45	9.74
StDDP9	5772	1137	ST4.03ch08	29755774..29761546	378	42,947.2	9.29
StDDP10	7418	972	ST4.03ch09	46690336..46697754	323	37,409.37	9.12

*CDS, coding sequence; pI, isoelectric point.*

### Gene Structure and Conserved Motif Analysis

**FIGURE 2:**
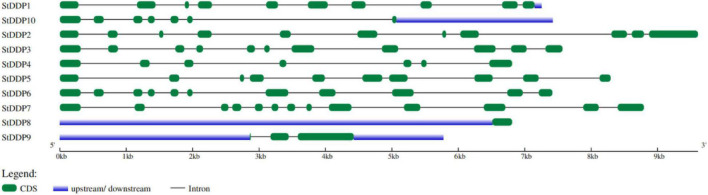
Distribution of exons, introns, and UTR (untranslated regions) in *StDDP* gene sequences.

The genomic DNA and coding DNA sequences were used for exon-intron structure analysis in *S. tuborosum*. The number, length, and distribution of introns-exons were not the same among all genes. For instance, *StDDP2* was the most extended, whereas *StDDP9* was the smallest gene. The number of *StDDP* exons ranged from 1 to 13. In our results, *StDDP8* has only one exon, whereas *StDDP7* has a maximum of 13 exons. However, some genes, such as *StDDP2*, *StDDP3*, and *StDDP6* have a similar number of exons. In addition, *StDDP1* and *StDDP5* have an equal number of exons but with different sequence lengths (Figure 2).

**FIGURE 3:**
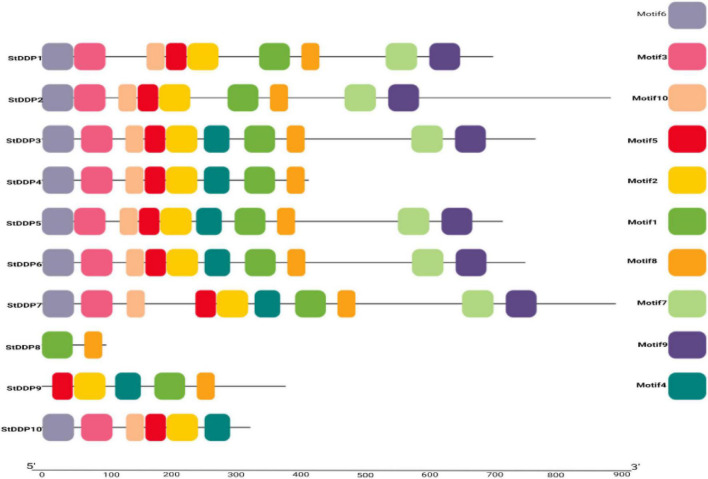
*De novo* MEME motifs’ distributions in the StDDP proteins.

The architecture of StDDP proteins was also investigated using DDP amino acids sequences. The MEME motif analysis identified several common and unique motifs in *StDDPs*. Commonly shared motifs tended to cluster in the same groups, indicating similar functions. The first motifs were observed in all *StDDPs*, except *StDDP10*, whereas the second motif was observed in most of the proteins except *StDDP8.* Motif 3 was absent in *StDDP8*. In summary, some motifs were family-specific, group-specific, clade, and taxa-specific. The length of motifs also varied; for example, the first and ninth motifs had 49 amino acids, the second, third, sixth, and seventh motifs had 50 amino acids and the eighth, tenth, and fifth motifs had 29 amino acids (Figure 3).

### Chromosomal Distribution and Cis Element Analysis

**FIGURE 4:**
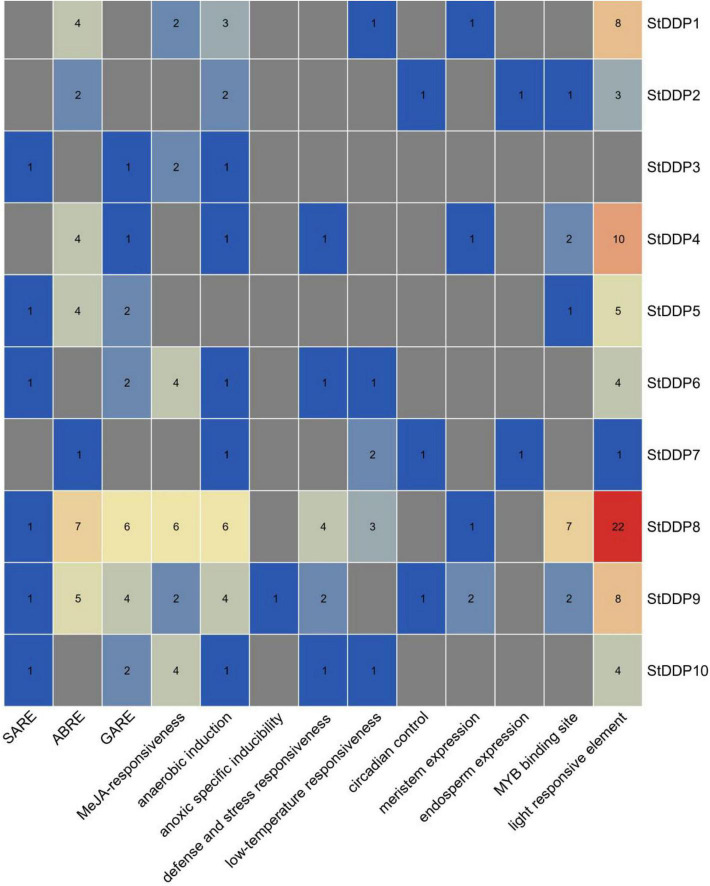
Predicted *cis*-acting elements in *StDDP* promoters. Promoter sequences (2,000 bp) of ten *StDDP* genes were analyzed by PlantCARE.

The numbers of *StDDP* genes were present unequally on chromosomes. The maximum number of *DDP* genes was observed on chromosome # 2 (four genes). A total of two *DDP* genes were present on chromosomes # 8 and 9. Only one gene was present on chromosomes # 4 and 10 (Table 1). The results of the *cis-*element analysis indicated that generally, the *cis*-acting elements were from growth and development, phytohormones, and stress-responsive classes. Light and MYB binding were the most enriched elements. Furthermore, anaerobic induction, stress-responsive components, and defense were also enriched in their promoters among stress-responsive components. The MeJRE and ABA response elements were enriched in phytohormones responsive elements. Consequently, it was observed that various *cis*-regulatory elements carried out *DDP* gene expression (Figure 4).

### Genes Duplications and Identification of Orthologous Gene Clusters

The rate of molecular evolution was measured for entire duplicated gene pairs with estimating the rate of Ka/Ks. The value of Ka/Ks > 1 was a positive selection factor, whereas the Ks/Ks < 1 were indicated as a purifying selection factor. Ka/Ks = 1 was indicated a neutral selection amongst the duplicated genes. Our results illustrated that the majority of the *DDP* duplicated genes tolerated the pressure of purifying selection during the duplication process, implying that the role of the *DDP* duplicated genes may not alter comprehensively in successive evolutionary processes. The time deviation among duplicated gene pairs was also approximated. The cosmic majority of *DDP* genes illustrated the Ks > 0.52 value, although the resulting time deviation may be greater than 100 MYA (millions of years ago). Surprisingly, in the present study, the *Ks* value for the duplicated gene (*StDDP1/StDDP2*) was 0.614, while the deviation time was 118.17 MYA (Table 2).

**TABLE 2:** Gene duplication and selection pressure of *StDDPs*.

Seq_1	Seq_2	Ka	Ks	Ka_Ks	Time (MYA)
*StDDP1*	*StDDP2*	0.215171807	0.614484198	0.350166542	118.1700381
*StDDP6*	*StDDP10*	0.02600552	0.037007255	0.702714102	7.116779808

*Ka, no. of nonsynonymous substitutions per nonsynonymous site; Ks, no. of synonymous substitutions per synonymous site; MYA, million years ago.*

**FIGURE 5:**
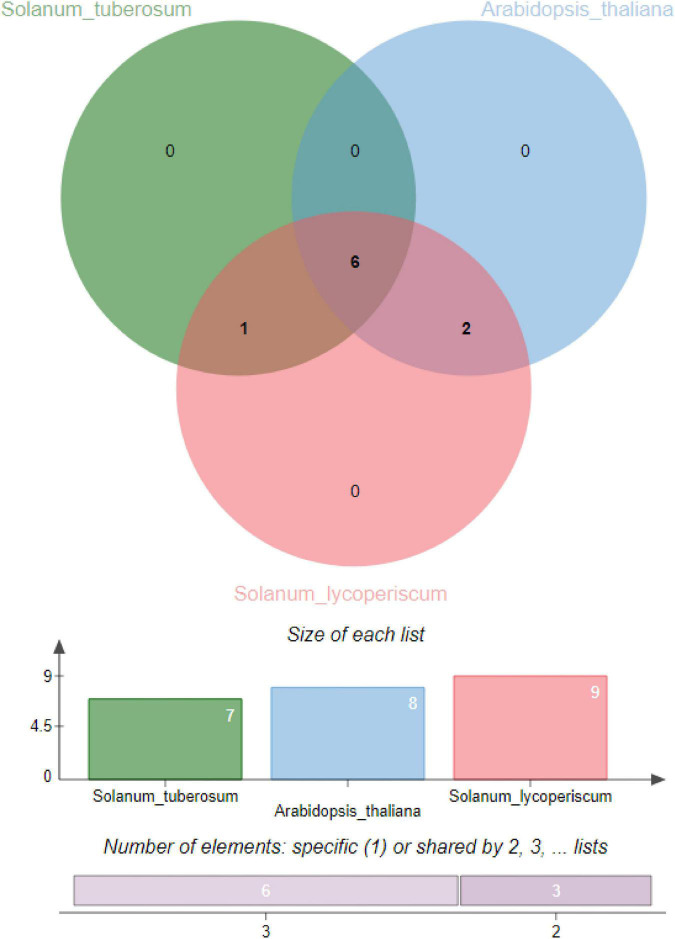
Identification of orthologous gene clusters in potato, tomato, and Arabidopsis genomes.

The corresponding evaluation was carried out to determine orthologous clusters of DDPs in potato, tomato, and Arabidopsis genomes. This will aid in analyzing processes related to polyploidization throughout the evolutionary period of the DDP gene family in the genome of *S. tuberosum*. Spotted orthologous gene clusters and their imbricated regions of *S. tuberosum* are depicted in Figure 5. The highest number of in-paralogous genes was noted in *S. lycopersicum*. Seven in-paralogous genes were identified in *S. tuberosum*, and eight in-paralogous genes were identified in *A. thaliana*.

### Comparative Synteny and miRNA Analysis

**FIGURE 6:**
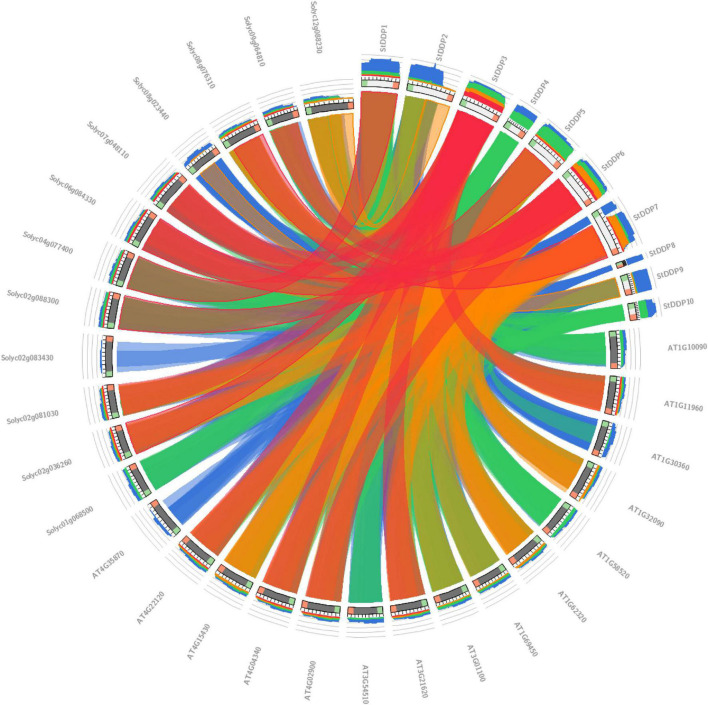
Synteny analysis of DDPs among potato, tomato, and Arabidopsis genomes.

The synteny analysis in *A. thaliana*, *S. lycopersicum*, and *S. tuberosum* illustrated the significant relationship between gene evolution, duplication, triplication, expression, and function. The *Solyc12g088230* gene sequence showed synteny in tomatoes with the *StDDP2* gene sequence of potato. Likewise, the potato gene *StDDP2* showed synteny with tomato *Solyc02g036260*. Potato *StDDP2* presented synteny with tomato *Solyc08g076310* (Figure 6).

In the previous few years, various studies have revealed miRNA-mediated regulation escorts stress responses in plants. The comprehensive information of entire miRNA targeted sites/genes is shown in Supplementary Table 2. These results illustrated that 10 members of the stu-miR395 targeted *StDDP4*. Three members of miR319-3p, stu-miR319a-3p, and stu-miR319b targeted two genes (*StDDP1* and *StDDP5)*. The stu-miR172a-5p targeted *StDDP7*, stu-miR172b-5p targeted *StDDP2* and stu-miR172d-3p targeted *StDDP1*. Furthermore, stu-miR8033-3p targeted *StDDP6. StDDP5, StDDP4, StDDP2*, and *StDDP1* genes were estimated to be targeted with more miRNAs. These results revealed that miRNAs have significant biological functions in the genome of the potato.

### Tissue-Specific Expression Profiling

**FIGURE 7:**
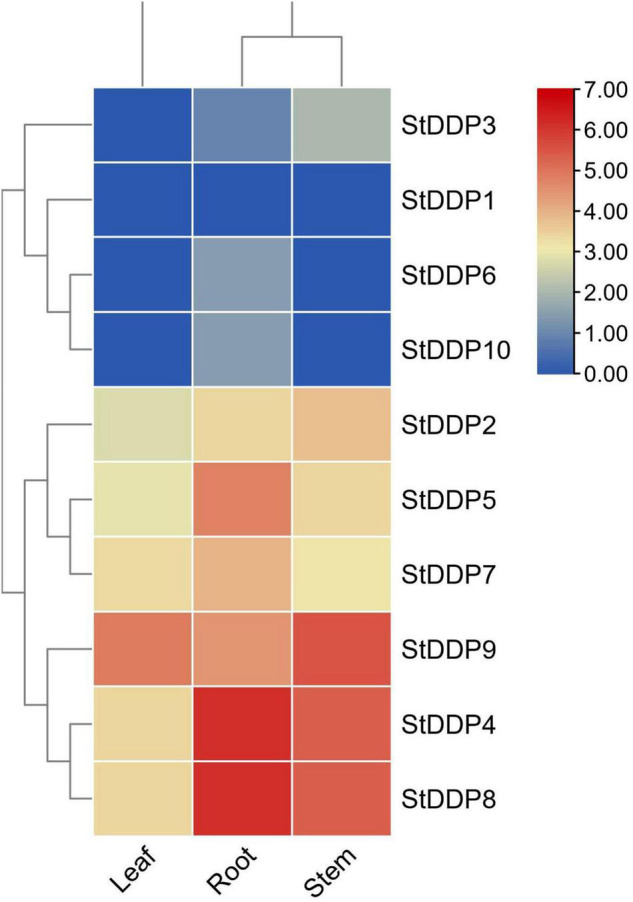
Expression profiling of *StDDP* genes in leaves, roots, and stems.

The expression profiling suggests that most *DDP* genes represented comparatively higher transcriptional abundance in roots than in stems and leaves. Though, some detected genes do not illustrate any expression, while others show a tissue-specific expression. Such as, *StDDP8* was expressed in leaf, root, and stem tissues. In our results, the *StDDP8* gene had high transcript abundance in the roots, and the *StDDP9* gene was highly expressed in stems and leaves (Figure 7).

### Expression Patterns of StDDP Genes in Response to Heat and Salt Stress

**FIGURE 8:**
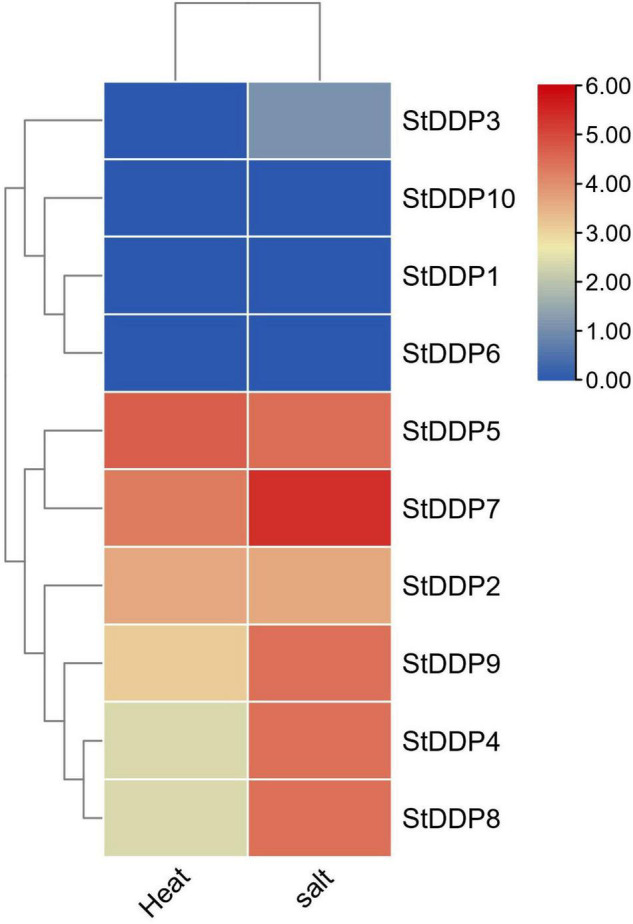
Expression profiling of *StDDP* genes in response to heat and salt.

Based on the transcription levels of *StDDPs* in potatoes under heat stress, we inferred that *StDDPs* might take part against potato heat stress. Six *StDDP* genes were expressed against heat treatment, although the transcription levels of *StDDP2*, *StDDP5*, and *StDDP7* are remarkably enhanced in potatoes after heat treatment (Figure 8). However, the expression level of *StDDP5* was observed to be higher than those of *StDDP2* and *StDDP7*. The expression of *StDDP4* was lower compared with those of other genes. A total of 7 *DDP* genes were upregulated against salt treatment, although the transcription levels of *StDDP5*, *StDDP7*, and *StDDP9* were remarkably enhanced in potatoes after salt treatment (Figure 8). The expression level of *StDDP7* was observed to be higher than those of StDDP5 and *StDDP9* in potatoes in response to salt treatment.

### Expression Patterns of StDDP Genes in Response to Phytohormones

**FIGURE 9:**
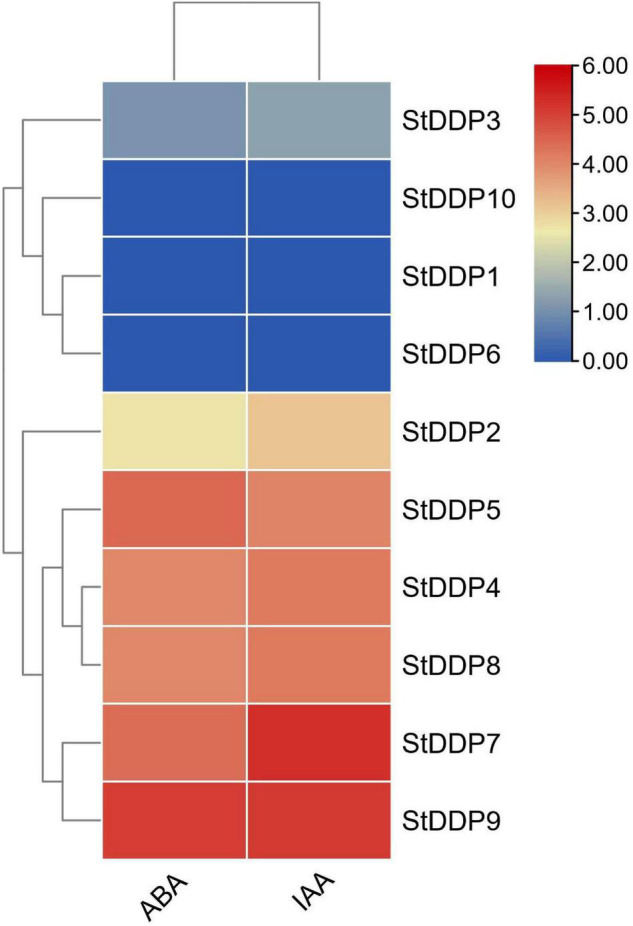
Expression profiling of *StDDP* genes in response to ABA and IAA.

Indole acetic acid (IAA) and abscisic acid (ABA) were selected to identify the *DDP* genes’ transcriptional responses toward hormones treatments. Leaf tissues were treated with ABA, and the expression pattern of all 10 genes was observed. A total of seven *DDP* genes showed expression against ABA treatment, and *StDDP9* showed higher expression compared to other *StDDPs*. Similarly, a total of seven *DDP* genes showed expression against IAA in treated leaves, and *StDDP7* showed higher expression compared to other *StDDPs* (Figure 9). These results indicated a strong correlation between gene clusters or their expression in IAA and ABA treatments.

### RT-qPCR Analysis

**FIGURE 10:**
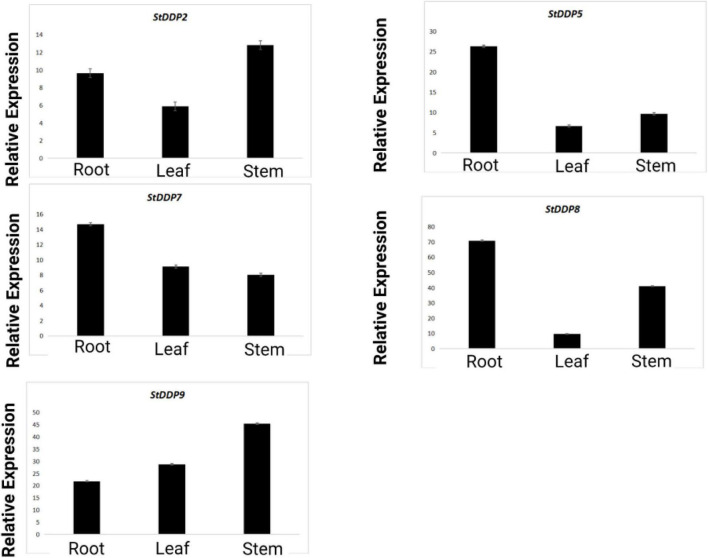
Real-time relative expressions of *StDDPs* in leaves, roots, and stems.

The RT-qPCR analysis also reported the expressions of the *StDDP2*, *StDDP5*, *StDDP7*, *StDDP8*, and *StDDP9* genes in leaves, roots, and stems. The comparative expression analysis pattern illustrated that *StDDP5*, *StDDP7*, and *StDDP8* have higher expressions in roots. Furthermore, *StDDP2* and *StDDP9* showed higher expression in stems than in roots and leaves. These results validate the RNA-Sequencing results. The higher expression in the roots demonstrates their strong association with the soil and environment (Figure 10).

## Discussion

Most plants bear destruction from biotic and abiotic stress agents (Zaynab et al., 2020), which influence their productivity and survival rate throughout their life cycle (Zaynab et al., 2018). *DDPs* are implicated in several regulatory processes, including development, growth, biotic and abiotic stresses (Zaynab et al., 2021e). Plants have evolved tolerance systems to change their cellular biochemistry and physiology during stress by changing gene expression (Zaynab et al., 2017). One of the domains known as DUF221 is considered a more conserved membrane-associated protein and acts to stimulate calcium osmoregulation through the cellular membrane (Hou et al., 2014). Thus far, the status of *DUF221* genes has been primarily unexplored. In this analysis, the DDP genes were observed in, salt, heat, and phytohormones stress, in keeping with previous identification in maize (Ding et al., 2019), rice (Li et al., 2015), and Arabidopsis (Yuan et al., 2014).

The structural identification of *StDDPs* is supportive during functional analysis.

All obtained *StDDP* genes were categorized into four phylogenetic groups. Similar phylogenetic grouping was also observed in other plant species. The comparative analysis of phylogeny illustrated that the organizations of *A. thaliana*, *S. lycopersicum*, and *S. tuberosum* proteins were relatively similar with each other in four clades, representing that all *StDDP* genes in representing groups may originate from a common ancestor. Previous studies have also reported the classification of *DDPs* into four clades in Arabidopsis (Yuan et al., 2014) and rice (Yuan et al., 2014; Li et al., 2015), and rice (Li et al., 2015).

Furthermore, evolutionary evidence found that exon-intron alignment affects gene family evolution (Moore and Purugganan, 2005). This is related to prior Scientific researches that several genes are subject to be kept in plants that could not have introns/short introns during the evolutionary process (Flagel and Wendel, 2009). In plants, gene expression levels are low with few and no introns (Mattick and Gagen, 2001). Moreover, a conserved gene configuration can allow a fast expression response toward an exogenous and endogenous gene stimulus (Jeffares et al., 2008). The structure analysis of the gene reported that the *StDDP* gene sequences illustrated a similarity in exon-intron number with analogous functional characters due to the origination of duplication course in evolutionary processes (Waqas et al., 2019). Some important factors, including gene distribution, genome size, and duplications, are involved in the genetic diversity of plants. However, the duplication factor of genetics has been extensively studied throughout the gene families’ expression origins, complexity, and evolutionary novelty. We also identified several duplication events in *StDDPs*, which played an important role in *StDDP* amplification. Gene duplication is an important event in expansion, diversification, and neofunctionalization (Lavin et al., 2005); correspondingly, the distribution and mapping of *StDDP* genes at the chromosomal level will support potato breeders in producing desired traits. *Cis*-element studies may illustrate an important foundation for functional analysis of the *StDDP* genes (Wen et al., 2021). Moreover, we also identified that all *StDDP* promoters hold multiple stress-responsive *cis-*elements, such as the low-temperature responsive elements, ABRE, the MeJA responsive elements, and the SA responsive elements (Raza et al., 2021a). These *Cis*-elements perform an essential function in stress response through stress-responsive gene regulation (Wu et al., 2014). Therefore, in *StDDP*s, these vital *Cis-*acting points propose their response to various environmental stress factors.

Over the past few years, through genome-wide examination, abundant miRNAs have been recognized in the potato to employ in different environmental factors. In addition obtained results reported that the DDPs in potatoes were targeted through miR172, miR8033, and miR319 members. These findings revealed that a particular gene may be regulated through multiple miRNAs. Some studies have reported the relationship between the *ZmDDP* (Ding et al., 2019) and *AtDDP* proteins (Zhao et al., 2015). The dynamic phytohormones and abiotic responsive expression patterns of *StDDP* genes are still ambiguous. The analysis of the expression pattern of *StDDP* genes supported us to understand their potential functions and propose a systematic base for future analysis. Therefore, *StDDPs*’ expression profiling and its validation are useful for deeper consideration of the potato genome. A current analysis presented a high transcript abundance of *DDPs* in plant roots; this finding was also established in a previous analysis (Yang et al., 2020) and helped in our results, in which *StDDP4*, *StDDP5*, and *StDDP8* showed high expression in roots.

The results of RT-qPCR indicated that *StDDP7* and *StDDP8* were particularly upregulated in roots. This suggests that potato *StDDP* genes play a vital function in development, growth and, diverse stress responses. So far, an increasing number of studies have illustrated the importance of *StDDPs* against various stresses (Zhao et al., 2021). Furthermore, in potatoes, *StDDP2*, *StDDP5*, and *StDDP7* were upregulated against heat stress. Though, the hormones can affect the physiological and biochemical reactions in plants during multiple signal transduction processes (Fatima et al., 2021). Moreover, IAA and ABA are important hormones in plant immunity. Numerous analyses have reported that *DDP*s are concerned with stress response and are involved in hormonal and developmental signaling (Waseem et al., 2021). To evaluate whether the *StDDPs* in potatoes were expressed by hormonal signaling, the potato leaves were treated with IAA and ABA, and gene expression was examined. After the ABA and IAA treatments, seven genes were influenced, indicating that various members of *StDDPs* played several roles in ABA- and IAA-induced immune response. During the ABA and IAA treatments, the upregulation of seven StDDP genes identified that ABA and IAA played important roles in the immune system, in accordance with previous studies. results (Waseem et al., 2021). The expression of genes and their clusters highlighted a strong correlation between gene groups and their analysis in various tissues during various stresses. This co-occurrence and co-expression illustrate their putative character related to plant adaptation under varied environmental stresses.

## Conclusion

Overall, a total of 10 *StDDP* genes were identified in *S. tuberosum*. The relative evolutionary analysis illustrated the existence of four main groups in the *DDP* gene family. Furthermore, the conserved structural and functional motifs were identified in *StDDPs*, through a slight change between groups and members. The high expression of *StDDPs* in roots demonstrated their significant role in plant–soil associations. The existing consequences present a profound understanding of main potato development and growth challenges under different stresses. In potatoes, *StDDP5* represented higher expression analysis in response to heat stress. During ABA and IAA treatments, the seven *StDDP* gene expressions demonstrated that ABA and IAA played essential roles in defense. Furthermore, the *StDDP9* gene showed higher expression against ABA treatment, and the *StDDP7* gene showed higher expression against IAA treatment.

## Data Availability Statement

The datasets presented in this study can be found in online repositories. The names of the repository/repositories and accession number(s) can be found in the article/Supplementary Material.

## Author Contributions

MZ, YS, and RA-Y performed the experiments and wrote the manuscript. HS, IA, MN, KK, and SA revised the manuscript. SL revised and supervised the manuscript. All authors contributed to the article and approved the submitted version.

## Conflict of Interest

The authors declare that the research was conducted in the absence of any commercial or financial relationships that could be construed as a potential conflict of interest.

## Publisher’s Note

All claims expressed in this article are solely those of the authors and do not necessarily represent those of their affiliated organizations, or those of the publisher, the editors and the reviewers. Any product that may be evaluated in this article, or claim that may be made by its manufacturer, is not guaranteed or endorsed by the publisher.

## Footnotes

1
http://www.arabidopsis.org/

2
https://phytozome.jgi.doe.gov/

3
https://web.expasy.org/protparam/

4
https://github.com/CJ-Chen/TBtools

5
http://meme-suite.org/

6
http://bioinformatics.psb.ugent.be/webtools/plantcare/html/

7
http://plantgrn.noble.org/psRNATarget/
